# Microcirculation changes in gingival tissue after ultrasonic tooth preparation in beagle dogs

**DOI:** 10.1590/1678-7757-2019-0145

**Published:** 2020-01-31

**Authors:** Masahiro TO, Masato MATSUO, Satoko WADA-TAKAHASHI, Shuta SUGIYAMA, Katsushi TAMAKI, Shun-suke TAKAHASHI

**Affiliations:** 1 Kanagawa Dental University Graduate School of Dentistry Department of Oral Science Kanagawa Japan Kanagawa Dental University, Graduate School of Dentistry, Department of Oral Science, Kanagawa, Japan.; 2 Kanagawa Dental University Graduate School of Dentistry Department of Critical Care Medicine and Dentistry Kanagawa Japan Kanagawa Dental University, Graduate School of Dentistry, Department of Critical Care Medicine and Dentistry, Kanagawa, Japan.

**Keywords:** Gingival microcirculation, Laser Doppler flowmetry, Resin cast, Scanning electron microscopy, Ultrasonic preparation

## Abstract

Ultrasonic wave technology is widely used during dental treatments. We previously demonstrated that this method protects the gingival tissue. However, the physiological change on the gingival microvasculature caused by this method remains unclear.

**Objective:**

The aim of this study was to investigate the relationship between the morphological and physiological effects on gingival microcirculation when preparing teeth, using the conventional dental turbine or ultrasonic method.

**Methodology:**

The lower premolar teeth of beagle dogs were prepared along the gingival margin by using a dental turbine or ultrasonic wave instrument. Gingival vasculature changes were investigated using scanning electron microscopy for corrosion resin casts. Gingival blood flow at the preparation site was determined simultaneously by laser Doppler flowmetry. These assessments were performed immediately (Day 0), at 7 days and 30 days after tooth preparation.

**Results:**

At day 0, in the turbine group, blood vessels were destroyed and some resin leaked. Furthermore, gingival blood flow at the site was significantly increased. In contrast, the ultrasonic group demonstrated nearly normal vasculature and gingival blood flow similar to the non-prepared group for 30 days after preparation. No significant alterations occurred in gingival circulation 30 days after either preparation; however, the turbine group revealed obvious morphological changes.

**Conclusions:**

Based on multiple approach analyses, this study demonstrated that ultrasonic waves are useful for microvascular protection in tooth preparation. Compared with a dental turbine, ultrasonic wave instruments caused minimal damage to gingival microcirculation. Tooth preparation using ultrasonic wave instruments could be valuable for protecting periodontal tissue.

## Introduction

Ultrasonic wave technology is commonly used during dental treatments. For example, an ultrasonic scaler is used for periodontal and endodontic treatment,^1^ whereas an ultrasonic knife is used during oral surgery.^2^ Tooth preparation is typically conducted 0.5 mm under the gingival margin. When using rotating instruments (e.g., dental air turbine), a part of the gingiva is damaged, thereby causing bleeding. Therefore, ultrasonic instrument is useful for tooth preparation since it maintains the margins in better condition.^3 , 4^ Furthermore, this instrument provides sufficient dentin-cutting capacity without injuring the marginal periodontium.^5^ We previously demonstrated that this method protects the microcirculatory system in the gingival tissue.^6^ However, its influence on the physiology of the gingival microvasculature remains unclear.

The gingival microvascular network has been investigated using animals in previous research.^7^ The formation of looping structures in gingival microcirculation could protect against bacterial invasion.^8^ In addition, the morphology of the microvasculature of gingival tissue has been observed by using the India ink method.^7 , 9^ The corrosion resin cast technique is widely available for observation of blood vessels in peripheral tissues, such as the gingival.^10 - 13^ This technique can clearly obtain an image of the vasculature, including capillaries, by using a scanning electron microscope (SEM). The resin cast accurately reproduces changes in gingival circulation.^14^

In addition, it has been demonstrated in human and animal studies that the gingiva blood flow can be changed by inflammation.^8^ The laser Doppler flowmetry (LDF) is a non-invasive method that can be used to estimate the hemodynamics of microcirculation, and is an accurate and reliable method for assessing other microcirculation characteristics. This approach is widely utilized in pharmacological and/or physiological areas of research involving allergy testing, wound healing, and dermatosis.^15^ While the skin is possibly the most studied organ, using LDF, it is also used to investigate internal organs such as the kidneys, liver, muscles, intestines, and brain.^16 - 21^ Furthermore, previous reports have demonstrated that LDF is highly reproducible and enables blood flow to be measured in various environments.^16 - 21^ Recently, the LDF has been applied to determine blood flow alterations with high reproducibility in the maxillofacial region, including the gingival mucosa, and gingival vascular function.^22 - 25^

The aim of this study was to determine whether ultrasonic preparation led to alterations in blood flow. We investigated the morphological and physiological effects of different tooth preparation methods — a conventional dental turbine or an ultrasonic instrument — on gingival microcirculation.

## Methodology

### Animal subjects and tooth preparation

**Figure 1:**
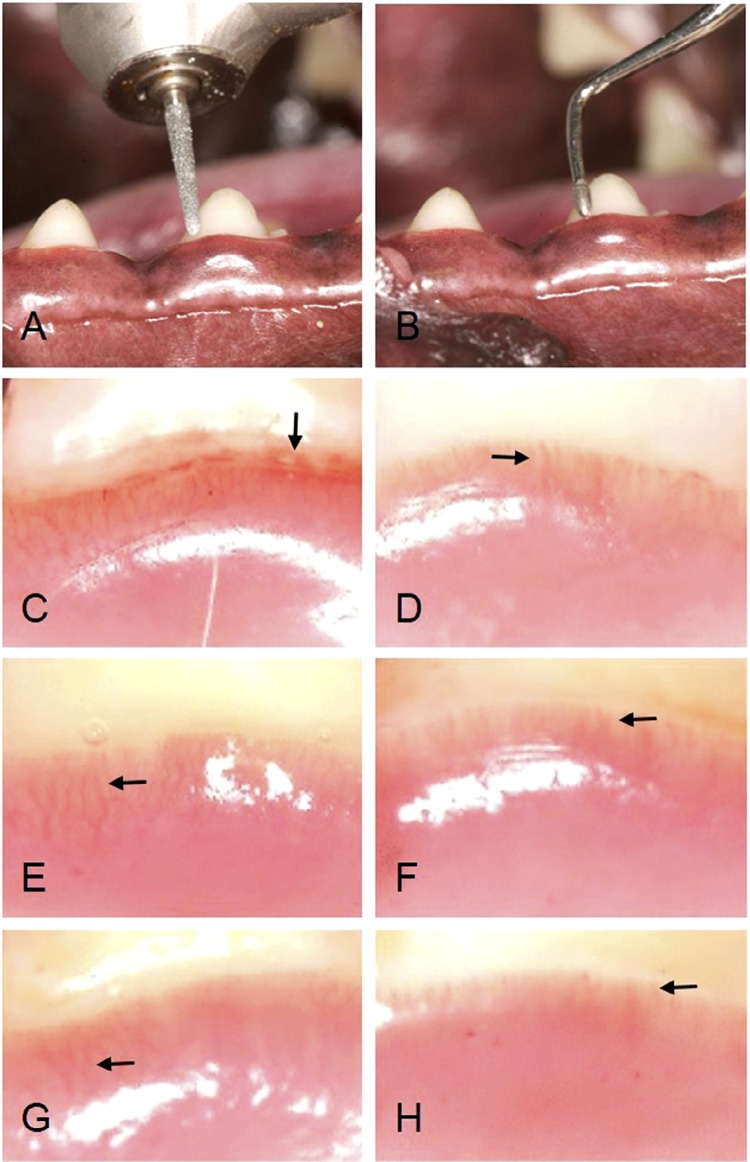
An oral view of the gingival blood vessels in the turbine group and ultrasonic group. A: The diamond bur and dental air turbine. The dentition on the one mandible was prepared by a diamond bur and dental turbine with water spray. B: An ultrasonic device using a diamond tip. The dentition on the mandible was prepared by ultrasonic waves using a diamond tip with water. C: On Day 0, in the turbine group, blood vessels are extended with bleeding (arrows). D: On Day 0, in the ultrasonic group, a vascular loop (arrows) was continuously formed along the finishing line. E: On Day 7, in the turbine group, blood vessels are extended (arrows). F: On Day 7, in the ultrasonic group, a vascular loop (arrows) was formed along the finishing line. G: On Day 30, in the turbine group, an extended vascular loop (arrows) is visible in the marginal gingiva. H: On Day 30, in the ultrasonic group, a vascular loop (arrows) was formed in the marginal gingiva

In this study, eight female beagle dogs (Oriental Kobo, Tokyo, Japan) had clinically healthy periodontal tissue; their teeth had undergone scaling 14 days before the surgery. The sample size for each group was six dogs — two dogs were operated in each day — for both two methods of tooth preparation. The control group (healthy gingiva) was composed by two dogs, which received no treatment. The teeth were prepared, as described in previous study.^6^ Briefly, under general anaesthesia with an intravenous injection of pentobarbital sodium (25 mg/kg, SOMNOPENTYL; Kyoritsuseiyaku Co., Ltd., Tokyo, Japan), the lower premolar teeth were prepared along the gingival margin. On one mandible, the premolar teeth were prepared with a diamond bur (SF102R, Shofu Inc., Kyoto, Japan) and a dental turbine (MIJET-T Yoshida, Tokyo, Japan) with water spray ( Figure 1A ); on the other mandible, the premolar teeth were prepared with ultrasonic waves (Suprasson P-max; Satelec, Merignac, France) using a diamond tip (FLT tip; Hakusui Trading Co., Osaka, Japan; Figure 1B ). To ensure that the two methods of tooth preparation were comparable, they were performed by the same dentist. To observe the gingival blood vessels in the healthy gingiva, the control group (female beagle dogs, Oriental Kobo) was not operated, aiming to observe the gingival tissue vasculature. The control animals were treated with chlorhexidine daily to control plaque during the experimental period. All the experiments with animals were conducted in compliance with the ARRIVE (Animal Research: Reporting of In Vivo Experiments) and the protocol was reviewed and approved by the Animal Care Committee of Kanagawa Dental University (Approval no. 10-0714, Yokosuka, Japan).

### Physiological procedure

Before conducting the morphological examination, gingival blood flow was determined at two points (mesial and centre) of eight lower premolar teeth at the same sites as tooth preparations using a dental turbine (four premolar teeth on one mandible) or ultrasonic waves (four premolar teeth on the other mandible). Moreover, gingival blood flow was determined in the non-prepared control animals at the equivalent sites. All gingival blood flows were estimated using an LDF meter (TBF-LN1; Unique Medical Co., Ltd., Tokyo, Japan) with a laser Doppler probe with 2.0 mm of diameter, and a calibration was performed before each analysis by the automatic calibration system according to the manufacturer’s instructions. These parameters were determined immediately (Day 0), 7 days, and 30 days after tooth preparation. The output signals from the flow meter were recorded on a computer hard disc and displayed simultaneously on the monitor. The recorded gingival blood flow was analyzed using data analysis software (Chart v 4.2; AD Instruments, Inc., Colorado Springs, CO, USA). The determined gingival blood flows were averaged for every bilateral dentition.

### Morphological procedure

After investigation of gingival blood flow at each time point, all animals were sacrificed by perfusion fixation under deep anaesthesia. Morphological procedures were conducted as described in a previous study.^6^ Vascular resin was injected on the day of the operation, as well as at 7 and 30 days of experiment. After perfusion fixation, the synthetic resin (Mercox; Ladd Research, Williston, VT, USA) was injected from the inferior alveolar arteries. The soft tissue was dissolved by 5% hypochlorous acid; all specimens were then washed thoroughly with water and freeze-dried. After specimens had been ion-coated with platinum palladium, they were examined using an SEM (JSM6301F; JEOL, Tokyo, Japan). To confirm morphological changes in the tooth surface state, hematoxylin and eosin staining was performed following the standard techniques and then, tissues were observed with a light microscope (Olympus Optical, Tokyo, Japan).

### Statistical analyses

Statistical analyses were performed using GraphPad Prism (version 6.05.; GraphPad Software, La Jolla, CA, USA). Values are expressed as the mean±standard error of the mean. Data were analyzed using the two-way (methods of preparation × days after operation) variance analysis, followed by the Tukey’s test. A P-value<0.05 was statistically significant.

## Results

### Oral view

On Day 0 of tooth preparation, bleeding occurred along the margin (arrows) in the turbine group. Moreover, blood vessels were extended ( Figure 1C ). In the ultrasonic group, a vascular loop (arrows) was formed along the finishing line ( Figure 1D ). On Day 7, blood vessels were extended in the turbine group ( Figure 1E ; arrows), whereas a vascular loop (arrows) was formed along the finishing line in the ultrasonic group ( Figure 1F ). After 30 days of tooth preparation, a vascular loop had formed in the marginal gingiva in both groups ( Figure 1G , H ); however, blood vessels were extended in the turbine group ( Figure 1H ; arrows).

Morphological observation with scanning electron microscopy and hematoxylin and eosin staining

**Figure 2:**
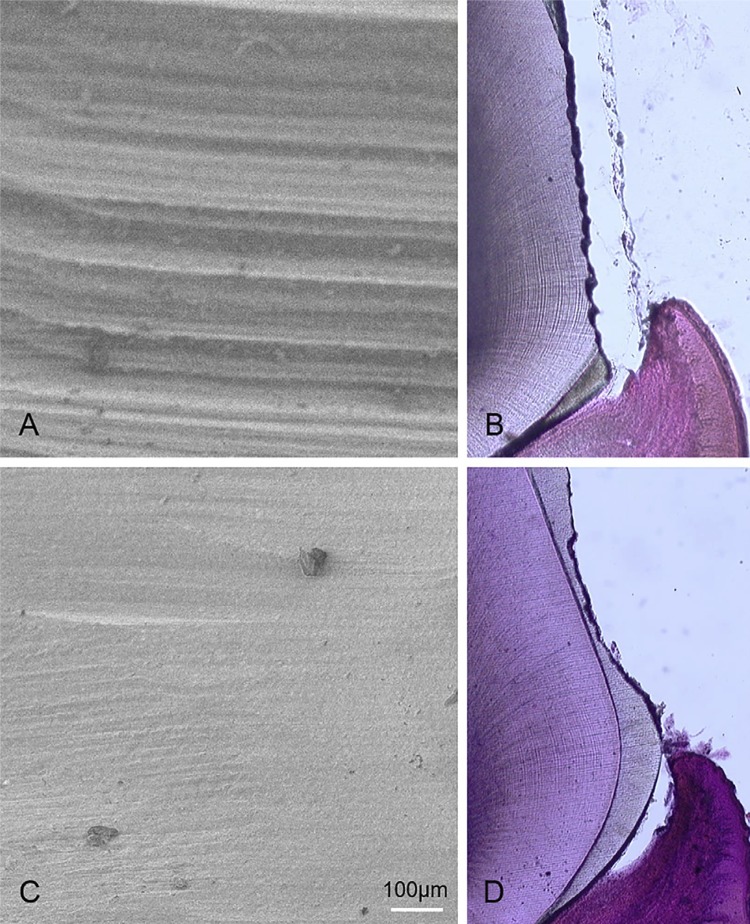
Scanning electron microscope (SEM) and hematoxylin and eosin staining observations of the prepared tooth surfaces. The cutting surface was observed using an SEM (A, C) and hematoxylin and eosin staining (B, D). A, B: Irregular and lateral stripes are visible in the turbine group. C, D: The surfaces are flat and smooth in the ultrasonic group

To confirm the state of the tooth surface, based on the preparation method, the cutting surface was observed using an SEM, haematoxylin, and eosin staining. In the dental turbine group, irregular and lateral stripes were observed ( Figures 2A and 2B ). However, in the ultrasonic group, flat and smooth surfaces were observed ( Figures 2C and 2D ).

### Resin casts of the gingival vascular network

**Figure 3:**
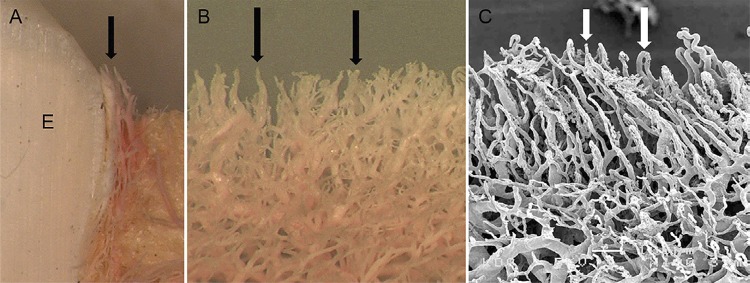
Resin casts of the gingival vascular network in the control group with healthy gingiva. A, B: Gingival blood vessels are attached to the tooth surface (indicated by “E”). Vascular loops are attached along the gingival margin (arrows). C: U-turn vascular loops converge at the gingival margin

The gingiva vasculature was visible in the control group ( Figure 3 ). A buccolingual section of the resin cast model was examined with a light microscope ( Figure 3A ). Gingival blood vessels were attached to the tooth surface (indicated by “E”) and vascular loops were visible along the gingival margin (arrows). On the buccal surface ( Figure 3B ), vascular loops were attached along the gingival margin (arrows). Using the SEM ( Figure 3C ), U-turn vascular loops converged at the gingival margin (arrows) and were composed by 10–15 µm capillaries and a deeper layer consisting of 50–100 µm main blood vessels.

### Day 0 of tooth preparation

**Figure 4:**
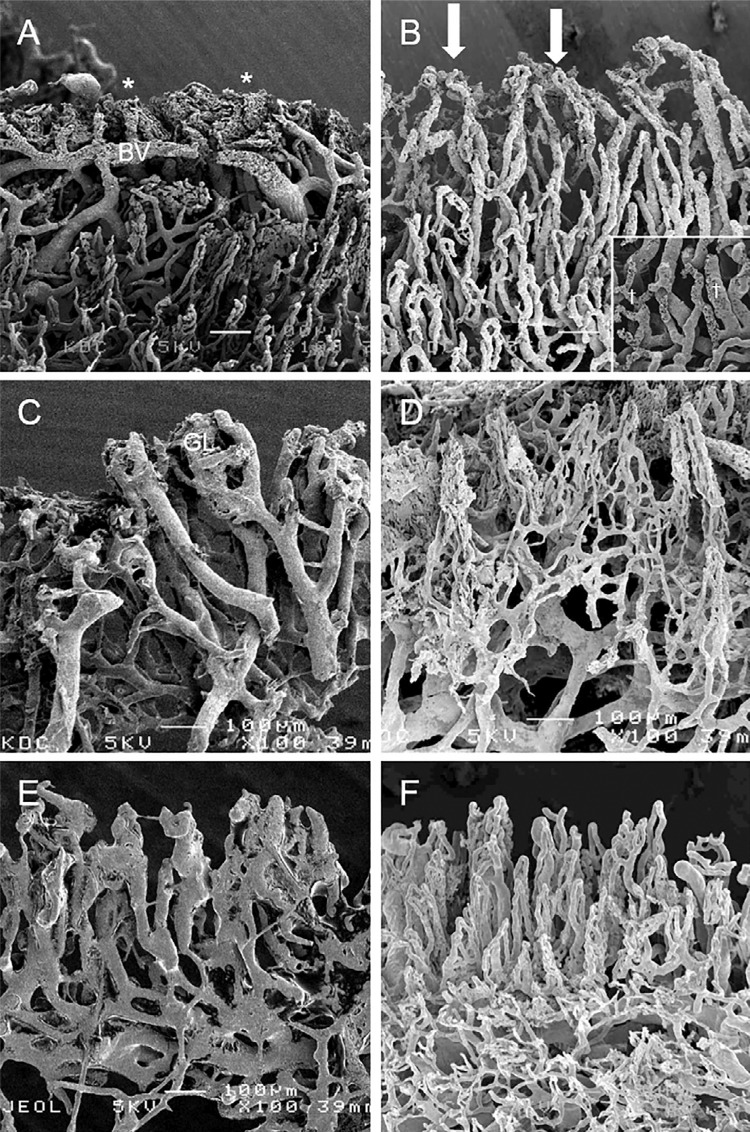
Vascular loops of the gingival margin in the turbine and ultrasonic group. A: On Day 0, in the turbine group, the injected resin flowed from the vessels (*), and main vessels (BV) in the deeper areas are on the gingival surface. B: On Day 0, in the ultrasonic group, the vascular networks are quite similar to those of the control group. U-turn loops were visible in the marginal gingiva (arrows). Round concavities (†) on the surface of blood vessels indicate the presence of red blood cells (square frame). C: On Day 7, in the turbine group, the vessels of the marginal gingiva were thicker in diameter (30–90 µm; GL) and glomerular loops were formed. D: On Day 7, in the ultrasonic group, thin U-turn loops exist in the marginal gingiva. These loops indicate the blood vessels regeneration . E: On Day 30, in the turbine group, blood vessels consisting of flat and thick loops are in the marginal gingiva. F: 30 days after tooth preparation in the ultrasonic group, vascular networks were arranged in a nearly normal manner

In the dental turbine preparation group ( Figure 4A ), the U-turn vascular loops of the gingival margin were destroyed by the turbine bur, and the injected resin flowed from the vessels [indicated by the asterisks (*)] along the margin. The main vessels in a deeper area appeared on the gingival surface. Regarding the remaining blood vessels, the U-turn vascular loops of the attached gingiva were visible on the buccal side. A dilated or large diameter vascular network was observed, which indicated that parenchymatous defects in the vascular loop of the marginal gingival were induced by turbine preparation.

In the ultrasonic group of tooth preparation ( Figure 4B ), the vascular networks quite resembled those of the control group. U-turn loops were visible in the marginal gingiva (arrows), although the diameters of the vessels were expanded. Round concavities [indicated by the daggers (†)] were visible on the blood vessels surface indicating the presence of red blood cells.

### Seven days after tooth preparation

In the dental turbine preparation group ( Figure 4C ), the vasculature was quite different from that of the control group. The vessels in marginal gingiva were thicker in diameter and formed glomerular loops. Most glomerulus-shaped gingival vascular loops disappeared and blood vessels had a relatively larger diameter. In the ultrasonic tooth preparation group ( Figure 4D ), thin U-turn loops were observed in the marginal gingiva. These loops indicated the regeneration of blood vessels.

### Thirty days after tooth preparation

In the dental turbine group, the blood vessels were creating U-turn loops in the marginal gingiva ( Figure 4E ), these vessels were flat and thick (diameter 50–80 µm). In the ultrasonic group ( Figure 4F ), most part of the vascular networks were arranged regularly. The marginal gingiva formed dense U-turn loops, which were similar to those in the control group (diameter 10–20 µm).

### Physiological observations: gingival blood flow evaluated with laser doppler flowmetry

**Figure 5:**
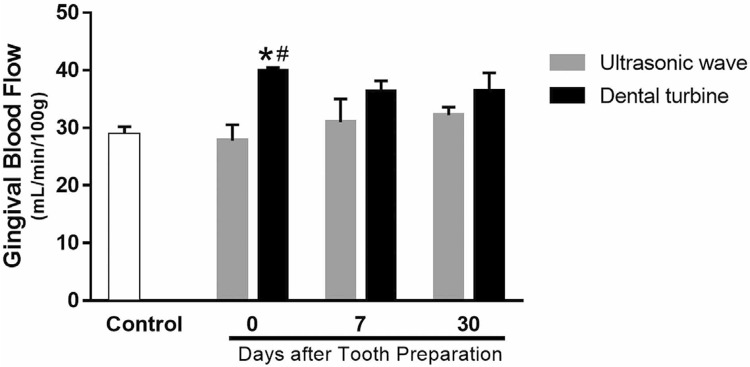
The effects of tooth preparation using ultrasonic or turbine on blood flow. The x axis represents the days after tooth preparation (ultrasonic: shaded column, turbine: closed column) and the y axis the gingival blood flow. Gingival blood flow was simultaneously determined at two points (mesial and centre) on each of the four lower premolar teeth and at the same site as the tooth preparation. These parameters were determined immediately (Day 0), at 7 days, and at 30 days after tooth preparation. Data are presented as the mean±the standard error of the mean (no.=6 in each method group) *P<0.05, versus the control group. #P<0.05, versus Day 0 of the ultrasonic tooth preparation

Gingival blood flow at the same site that the tooth preparation was significantly increased in the dental turbine group immediately (Day 0, P<0.05) and were recovered to same level observed in the control group after 30 days of tooth preparation ( Figure 5 ). Otherwise, the ultrasonic wave group had no significant alteration in gingival blood flow in the preparation site at Day 0 nor 30 days after preparation ( Figure 5 ). To record, the values of gingival blood flow were as follows: turbine group at 7 days, 36.32 mL/min/100 g; turbine group at 30 days, 36.45 mL/min/100 g; control group, 28.82 mL/min/100 g; ultrasonic group at 0 days, 27.75 mL/min/100 g.

## Discussion

In this study, the gingival microcirculation changes that were induced by different tooth preparation methods were assessed by morphological and physiological alterations. In the chronological assessment of tissue damage after turbine or ultrasonic preparation, morphological alterations in the vasculature due to inflammation were apparent, although cases may occur without remarkable traits in gingival blood flow. Moreover, there may be no differences in blood flow among healthy, inflamed and in healing process gingiva. Our previous study recently proved that functional analyses using a single index may not be accurate and should be accompanied by appropriate morphological analyses.^22^ In this study, we demonstrated, by using morphological and physiological approach, that ultrasonic wave instruments cause minimal damage to gingival microcirculation, compared with a dental turbine.

Injection methods are typically used to observe the vasculature.^6 , 26^ The arterioles in the sulcular and junctional epithelium (JE) form a capillary network.^6^ Because of the vascularization importance for tissues regeneration, it is essential to protect the marginal gingiva during tooth preparation.^6^ Resin leakage, which indicates damaged blood vessels,^6^ was widely visible at the marginal gingiva and the JE. This situation represented widespread gingival bleeding observed at Day 0 as well as Day 30, after turbine preparation. Morphological alterations by turbine preparation were based on the finding that gingival blood flow increased immediately after this method (Day 0 in Figure 5 ).

Similarly, the gingival blood flow increased on day 7; however, it was not significantly different from the control group ( Figure 5 ). Increased gingival blood flow after turbine preparation was reduced on day 30; however, the gingival blood flow tended to be higher after turbine preparation than after ultrasonic preparation ( Figure 5 ). These findings indicate that the vascularization of the gingival margin network is disrupted by turbine preparation.

However, a resin leak was detected in the JE, which had not been in contact with the bur. A previous study reported that blood vessels showed signs of acute inflammation caused by heat in a tooth preparation performed without water.^27^ Similar to the typical initial pathological process of acute inflammation, a tooth preparation causes dilation of the venular capillaries and the formation of an endothelial gap by inflammatory cytokines. Previous SEM studies have shown that accelerated vascular permeability induces a synthetic resin leak.^28 , 29^ Moreover, a dental turbine induces pyrexia; therefore, a resin leak can occur in the endothelium because of this gap formation.^30^ Furthermore, a large number of the capillaries in the gingival tissue are fenestrated blood vessels,^6^ which induce a frequent exchange of fluid. After 30 days of preparation, glomerular loops were in the marginal gingiva, although their structure differed from the normal structure. This finding suggests that additional recovery time is needed.

The ultrasonic preparation group had no bleeding in the marginal gingiva, and the turbine preparation group had no vascular destruction. However, some irregularities regarding the resin cast in the gingival surface were detected in the turbine group. Notably, the vascular loop at the marginal gingiva was maintained after ultrasonic preparation ( Figure 4B ). The increase recorded in blood flow after turbine preparation did not occur immediately after ultrasonic preparation; however, gingival blood flow tended to decrease slightly through the vessels because of damaged and enhanced permeability after ultrasonic preparation ( Figure 4B and Figure 5 ). New vascular vessels begin to grow again 14 days after the mechanical obstacles, including tooth preparation.^6 , 28 , 31^ . Normal vascular arrangement was visible in the marginal gingiva after 30 days ( Figure 4F ). Severe morphological and circulatory alterations observed after turbine preparation did not occur immediately in the ultrasonic preparation ( Figure 4B ), and those occurred changes, nearly recovered to the normal levels on day 7 ( Figure 4D and Figure 5 ). These findings suggest that ultrasonic preparation does not disturb the gingival vasculatore, its circulatory systems nor induce the thermal damage that occurs after turbine preparation.

A limitation of this study is that no significant differences were observed between groups on days 7 and 30 after tooth preparation. This study demonstrates the safeness of ultrasonic preparation, and the findings are similar to those of a previous study.^6^ Furthermore, the operability and clinical applicability of ultrasonic preparation were similar to those of dental turbine. Possible damage induced by ultrasonic preparation may have a minimal effect on the condition of periodontal tissues. It is well-known that the retention of a dental prosthesis on a dental abutment after tooth preparation is closely related to the periodontal status. Therefore, future studies are needed to investigate whether different methods affect the long-term retention of dental prostheses.

This study was conducted on beagle dogs with clinically healthy periodontal tissue. The condition of gingival tissue can be changed by factors such as age, progression of periodontitis, and cardiovascular disease.^32^ Moreover, inflammatory mediators are involved in the homeostasis of the marginal gingiva.^33^ Future studies are needed to clarify the effect of these factors on periodontal tissue.

## Conclusions

By using multiple approach analyses, this study demonstrated that ultrasonic waves are useful for tooth preparations to minimize damage to the gingival tissue. Protecting the marginal gingiva during tooth preparation is essential since vascularization is important for tissue regeneration. Destruction of the JE causes periodontal disease, which suggests that tooth preparation using ultrasonic wave instruments is essential for protection of the periodontal tissue.
